# Antibody Response to SARS-CoV-2 in the First Batch of COVID-19 Patients in China by a Self-Developed Rapid IgM-IgG Test

**DOI:** 10.3389/fcimb.2022.915751

**Published:** 2022-05-27

**Authors:** Yiyi Pu, Youhong Weng, Yahan Wu, Fei Gao, Xiaojun Zheng, Xianqin Xiong, Hangjun Lv, Qingming Kong

**Affiliations:** ^1^ School of Laboratory Medicine and Bioengineering, Hangzhou Medical College, Hangzhou, China; ^2^ Institute of Parasitic Diseases, Hangzhou Medical College, Hangzhou, China; ^3^ Department of Research and Development, Hangzhou AllTest Biotech Co., Ltd, Hangzhou, China

**Keywords:** COVID-19, SARS-CoV-2, nucleocapsid protein, antibody profile, serologic test, lateral flow chromatographic immunoassay

## Abstract

It has been over two years since the COVID-19 pandemic began and it is still an unprecedented global challenge. Here, we aim to characterize the antibody profile from a large batch of early COVID-19 cases in China, from January – March 2020. More than 1,000 serum samples from participants in Hubei and Zhejiang province were collected. A series of serum samples were also collected along the disease course from 70 patients in Shanghai and Chongqing for longitudinal analysis. The serologic assay (ALLtest) we developed was confirmed to have high sensitivity (92.58% - 97.55%) and high specificity (92.14% - 96.28%) for the detection of SARS-CoV-2 nucleocapsid-specific antibodies. Confirmed cases found in the Hubei Provincial Center for Disease Control and Prevention (HBCDC), showed a significantly (p = 0.0018) higher positive rate from the ALLtest than RNA test. Then, we further identified the disease course, age, sex, and symptoms that were correlating factors with our ALLtest results. In summary, we confirmed the high reliability of our ALLtest and its important role in COVID-19 diagnosis. The correlating factors we identified will require special attention during future clinical application.

## Introduction

Since December 2019, cases of pneumonia with unknown etiology started to be reported in Wuhan city, Hubei province of China (World Health Organization, 2020a). Shortly after this, a new type of coronavirus was isolated and named the novel severe acute respiratory syndrome coronavirus 2 (SARS-CoV-2). With a rapid increase of case numbers caused by SARS-CoV-2, World Health Organization (WHO) declared Coronavirus disease 2019 (COVID-19) as a pandemic in March 2020 (World Health Organization, 2020b). Various measures have been taken, such as city lockdowns, travel restrictions, and mandatory mask wearing. Unfortunately, COVID-19 still quickly spread and recent estimates show that over 489 million cases and over 6 million deaths have been reported worldwide (World Health Organization, 2022).

RNA tests have long been considered as the gold standard, due to its direct viral detection. However, increasingly more shortcomings have been noticed which include, but not limited to, the following: 1) high false negative rate (Kucirka et al., 2020) which lead to unreliable results and repetitive tests, and 2) test results that depend on location (e.g. oropharynx, nasopharynx) (Wikramaratna et al., 2020). Under such circumstances, antibody tests with their unique features can support RNA test results and largely accelerate the detection speed (Yuce et al., 2021). More importantly, an antibody test is able to show the infection process (Yuce et al., 2021). Among all commonly used antibodies tests, the lateral flow immunoassay (LFIA) test has outstanding advantages such as being rapid and suitable for home-testing.

Although it has been over two years since the first COVID-19 outbreak, there is still strong need to look back to the very beginning and find useful information for future epidemic prevention and control. Therefore, to exhibit reliable results, we summarized and reported antibody data of more than 1,000 participants from five centers during the first three months of 2020. Here, using our self-developed rapid antibody test (ALLtest), we characterized serum antibody from multiple aspects.

## Materials and Methods

### The Design of Our Rapid Antibody Test (ALLtest) for COVID-19

**Figure 1:**
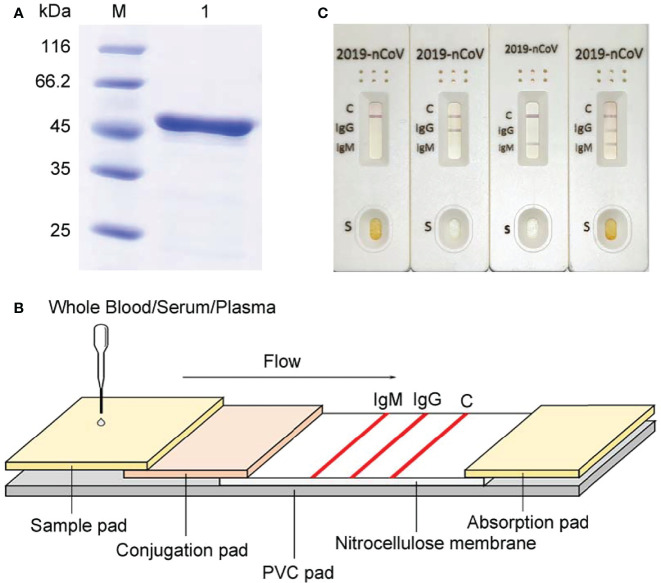
Development of our rapid SAR-CoV-2 antibody test (ALLtest). **(A)** Purification of COVD-19 nucleocapsid. M: markers; 1: recombinant SARS-CoV-2 nucleocapsid. **(B)** Schematic illustration of ALLtest. **(C)** Typical testing results of ALLtest (from left to right: negative, IgG positive, IgM positive, IgG&IgM positive).

Here we developed a SARS-CoV-2 IgG/IgM Rapid Test Cassette which is a qualitative membrane-based immunoassay for the detection of IgG and IgM antibodies to SARS-CoV-2 nucleocapsid in whole blood, serum, or plasma specimen. The COVID-19 nucleocapsid gene was synthesized according to the severe acute respiratory syndrome coronavirus 2 isolate Wuhan-Hu-1 (NC_045512.2) and then amplified by the polymerase chain reaction (PCR) with designed primers (Pu : GCCGGATCCATGTCTGATAATGGACCCCAAAA; Pd : GCCGTCGACAGGCCTGAGTTGAGTCAGCAC). The PCR products were cloned into the pET28a plasmid vector and expressed in BL21 *E. coli* cells (induced by 1mM IPTG, 37°C, 250rpm, 5h). Ni-NTA column purification was performed on 50ml bacterial solution to elute target proteins with different concentrations of imidazole. Then the expression of SARS-CoV-2 nucleocapsid was confirmed by 12% sodium dodecyl sulfate-polyacrylamide gel electrophoresis (SDS-PAGE) (stained with coomassie brilliant blue) (**Figure 1A**).

The entire package includes test cassettes, droppers, package insert, and buffer. The schematic illustration of our rapid SAR-CoV-2 antibody test cassette is summarized in **Figure 1B**. Our test cassette consists of a PVC pad (Hangzhou Ruijian Technology Co. Ltd), a sample pad (Ahlstrom Filtration LLC), a conjugation pad (Ahlstrom Filtration LLC), an absorption pad (Suzhou Equation Technology Materials Co. Ltd), and a nitrocellulose (NC) membrane (Sartorius stedim biotech). On the top of the PVC pad, after adding the sample and buffer onto the sample pad (combined with COVID-19 IgG & IgM antibodies), liquid flows from left to right going through the following: 1) conjugation pad combined with COVID-19 nucleocapsid antigen conjugate and mouse IgG conjugate, 2) NC membrane combined with specific antibodies on each line (IgM line - anti-human IgM antibody, IgG line - anti-human IgG antibody, control line - anti-mouse IgG antibody), and 3) absorption pad. Typical testing results are illustrated in **Figure 1C**. Results without control line were identified as invalid.

### Patient and Sample Collection

This study enrolled a total of 1,158 participants from five centers in China (The First Affiliated Hospital - Zhejiang University School of Medicine, Hubei Provincial Center for Disease Control and Prevention, The Third Hospital of Wuhan, Shanghai Public Health Clinical Center, Chongqing Public Health Medical Center), including 616 confirmed COVID-19 patients and 542 people with a normal health examination (basic information of all participants shown in **Table 1**). In HBCDC, some COVID-19 patients were confirmed of suspected cases by chest computerized tomography (CT) scan. First-time blood samples and throat swab samples were collected for all participants from January–March 2020. For all enrolled participants, age, sex, sampling time, and clinical diagnosis information were acquired from clinical records. Also, disease related information (e.g., disease onset time, disease severity) of all enrolled COVID-19 patients were also recorded. This study was approved by Ethical Committee of the First Affiliated Hospital - Zhejiang University School of Medicine (No. 2020-43), Hubei Provincial Center for Disease Control and Prevention (No. 2020-005-01), The Third Hospital of Wuhan (No. QX2020-002), Shanghai Public Health Clinical Center (No. YJ-2020-E027-01) and Chongqing Public Health Medical Center (No. 2020-048-01).

**Table 1:** Summary of participant information from five centers.

	Sum	Sex		Age range	Diagnosis	
		Female	Male		Y	N
**FAHZU (Pre-test)**	32	17	15	17-96	22	10
**FAHZU**	360	142	218	2-102	64	296
**HBCDC**	352	157	195	11-90	256	96
**WHSYY**	344	167	177	7-98	204	140
**SHAPHC**	30	15	15	20-76	30	0
**CQGWZX**	40	21	19	20-74	40	0

### Antibody Test and qRT-PCR Assay

Blood specimens were collected from all participants. Then 10 μl serum and 2 drops of buffer were transferred to the specimen well of ALLtest and results showed in about 10 minutes. Also, all participants underwent throat swab sampling and quantitative reverse transcription PCR (qRT-PCR) assay. Total viral RNAs were extracted from throat swabs by the MagNA Pure 96 (Roche, Basel, Switzerland) followed by SARS-CoV-2 test by a commercial kit (BoJie, Shanghai, China).

### Statistical Analysis

The clinical value of our ALLtest was evaluated by sensitivity and specificity. McNemar’s Chi-squared Test was conducted to compare the positive rate between ALLtest and RNA results. Pearson’s Chi-square test, Fisher’s exact test, T test, and Wilcoxon test were applied for categorical variables, categorical variables with insufficient sample size, normally distributed continuous variables, non-normally distributed continuous variables, or ordered categorical variables, respectively, to identify factors correlated with test results. Continuous variables and ordered categorical variables (e.g., age, disease course, severity) were analyzed by comparing the difference between test positive and negative groups. Those variables were identified to be correlating factors with significant difference. All statistical analyses were conducted by R (3.6.0).

## Results

### Sensitivity and Specificity of Our Rapid Antibody Test

Right after the development of the ALLtest, we preliminarily evaluated it by testing a small number of samples from FAHZU. Results showed high sensitivity and specificity (**Table 1S**. sensitivity = 100%, specificity = 90%). After verifying the above preliminary results, we further collected 1,056 samples from three centers (FAHZU, HBCDC and WHSYY, >340 samples from each center). Again, our results showed high sensitivity and specificity of the ALLtest (**Table 2**. FAHZU: sensitivity = 96.88%, specificity = 96.28%; HBCDC: sensitivity = 92.58%, specificity = 93.75%; WHSYY: sensitivity = 97.55%, specificity = 92.14%). Based on disease course information, we further researched detailed sensitivity. We found the sensitivity of the ALLtest was relatively consistent within 56 days of disease development (**Table 2S**), with the lowest sensitivity to be 96.83% in FAHZU, 84.62% in HBCDC, and 93.10% in WHSYY. The low sensitivity (84.62%) of HBCDC was due to 4 negative tests out of 26, with more than 28 days of disease development. With such a small sample size, unstable results could be easily obtained.

**Table 2:** Sensitivity and specificity of ALLtest in official test.

	FAHZU		HBCDC		WHSYY	
	Clinical confirmed	Clinical excluded	Clinical confirmed	Clinical excluded	Clinical confirmed	Clinical excluded
**Sample size**	64	296	256	96	204	140
**IgM positive**	34	8	148	5	101	6
**IgG positive**	61	5	235	6	195	5
**IgM/IgG positive**	62	11	237	6	199	11
**Sensitivity**	96.88%		92.58%		97.55%	
**Specificity**		96.28%		93.75%		92.14%

Clinically confirmed and clinically excluded cases were determined by positive and negative qRT-PCR result respectively.

**Figure 2:**
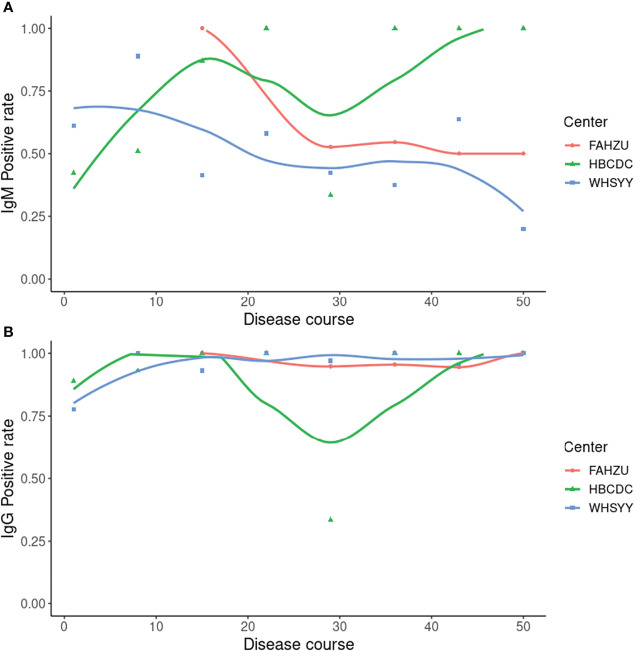
Positive rate of IgM and IgG by ALLtest. **(A)** Positive rate of IgM along the disease course; **(B)** Positive rate of IgG along the disease course. Curves were drawn by local polynomial regression fitting.

Sensitivity and specificity, noted above, were based on a combination of IgM and IgG results (negative result: negative results in both IgM and IgG; positive result: positive result in either IgM or IgG). As these are two kinds of immunoglobulin, it is also meaningful to look into the positive rate of IgM and IgG separately (**Figure 2**). As the less stable one, IgM showed a changing positive rate. In FAHZU and WHSYY, IgM, in general, showed only a moderate positive rate. But an increasingly higher positive rate of IgM was shown by HBCDC along the disease course. The positive rate of IgG remained high along the disease course in all three centers.

### Sensitivity Between Our Rapid Antibody Test and RNA Test

In all 256 clinical confirmed cases from HBCDC, there were 44 false negatives from the RNA test. From the ALLtest, 42 out of these 44 cases were identified with positive results by the IgM/IgG test. Therefore, we analyzed data from HBCDC by McNemar’s Chi-squared Test and found significantly higher positive rate of the ALLtest (92.58%) than the RNA test (82.82%) for all clinical confirmed cases (p = 0.0018, **Table 3S**).

### Factors Correlated With Antibody and RNA Test Results

**Figure 3:**
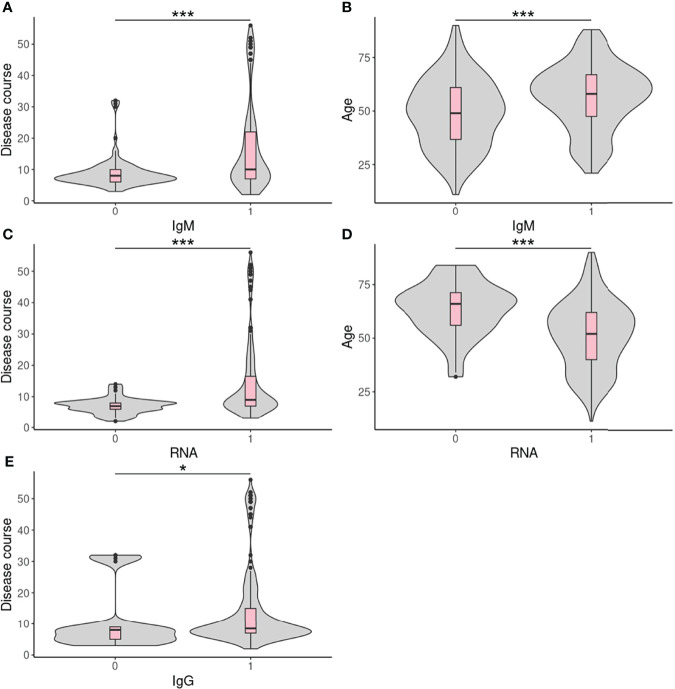
Continuous correlated factors in HBCDC. **(A–E)** Boxplots of continuous correlated factors in HBCDC. 0: negative result; 1: positive result. *p < 0.05, ***p < 0.001.

We attempted to identify factors correlated with test results in different centers by suitable statistical tests. No correlating factors were found in FAHZU (**Table 4S**). Disease course (sampling time - disease onset time) and age were found to be significantly correlated with IgM and qRT-PCR results in HBCDC (**Table 4S**; **Figure 3**). Also, disease course was correlated with IgG in HBCDC (**Table 4S**; **Figure 3**). In WHSYY, sex and symptoms were found to be correlated with IgM (**Tables 4S, 5S**).

### Antibody Dynamic With the Progress of Disease

**Figure 4:**
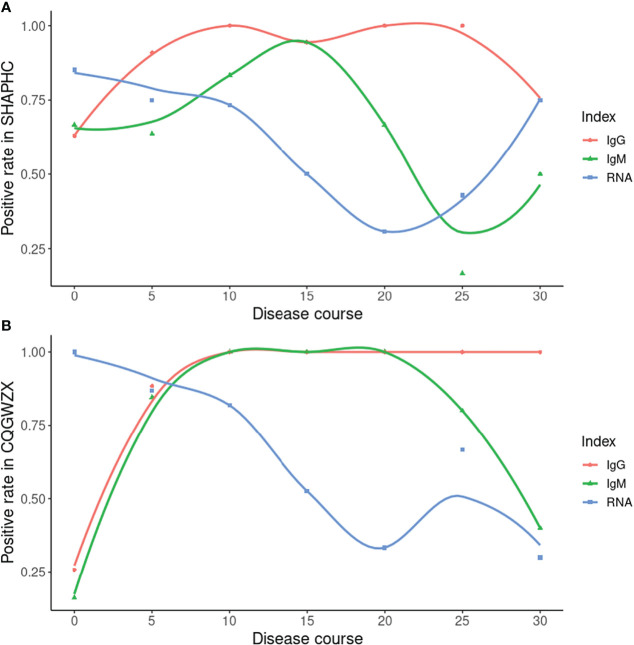
Time-series data from SHAPHC and CQGWZX. **(A)** Time-series data of 30 COVID-19 patients from SHAPHC; **(B)** Time-series data of 40 COVID-19 patients from CQGWZX. X axis: starting time point of 5 days period (0 means 0-4 days, 5 means 5-9 days and so forth, except for 30 which means all 30+ days result). Curves were drawn by local polynomial regression fitting.

We performed multiple tests for each confirmed COVID-19 patient from two centers (Shanghai Public Health Clinical Center, Chongqing Public Health Medical Center) along the disease course. Results of both centers were largely consistent. Within the early stage, the RNA test possessed a relatively high positive rate but was followed by a sharp decline. The high IgM positive rate only arrived at about 15 days after onset. The high IgG positive rate was reached about 10 days after onset and was maintained thereafter (**Figure 4**).

## Discussion

**Figure 5:**
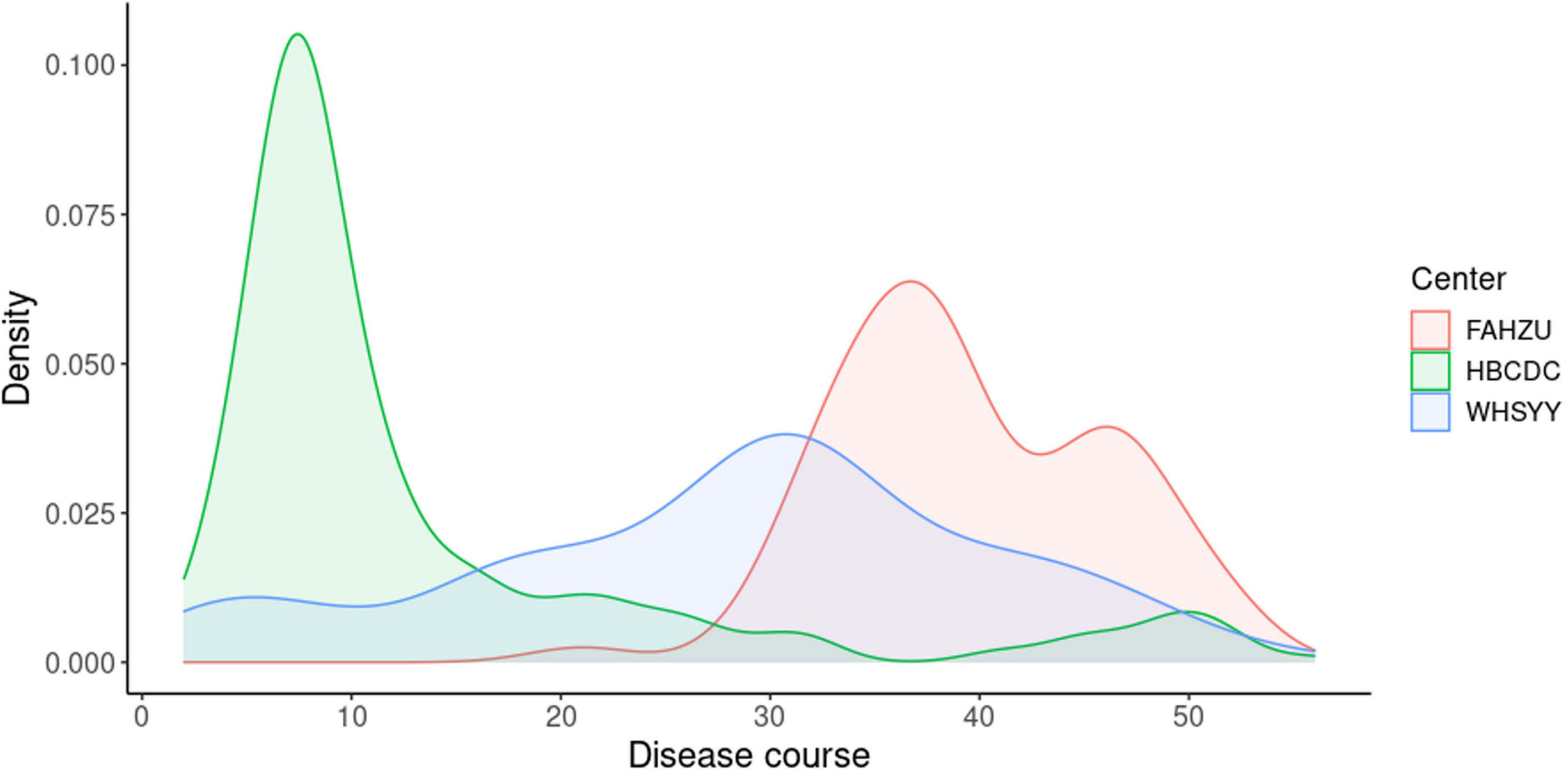
Distribution of disease course in three centers.

As two main types of antibodies, IgM and IgG show different patterns during the viral infection. IgM appears first but last for a limited amount of time (Galipeau et al., 2020). Conversely, IgG appears relatively late and last for a long time (Galipeau et al., 2020). Therefore, combining the results of both IgM and IgG, we are able to more sensitively detect SARS-CoV-2 infection than IgM or IgG alone. To get reliable results when using ALLtest, we depended on combined results (IgM/IgG). As a test developed for more than one year, our ALLtest has been evaluated by other researchers for different purposes. Pérez-García F et al., found the ALLtest reliable in detecting SARS-CoV-2 infection two weeks from onset (Perez-Garcia et al., 2020). Some other groups also used the ALLtest mainly in multiple method comparisons (Sacristan et al., 2020; Serrano et al., 2020; Wu et al., 2020). However, all of the above studies relied on a small number of samples. Therefore, we evaluated the ALLtest with a much larger group of serum samples and based on high sensitivity and specificity, our results indicate that the ALLtest might be more reliable than previously reported. We further looked into IgM and IgG results separately and found inconsistent results among centers. Results from all three centers agreed that IgG was a more reliable index in identifying SAR-CoV-2 infection. As for IgM, inconsistent results were largely derived from different distribution of disease courses (**Figure 5**). In HBCDC, cases were concentrated on early stages (~10 days after onset) and results of IgM were reliable only during such stages (**Figure 2**). Result filtration should also be applied in FAHZU and WHSYY, with cases mainly from later stages (~30-35 days after onset). After combining the above results from the three centers, as a whole, IgM showed only a moderate positive rate (**Figure 2**).

As the gold standard, the RNA test has been widely used in SARS-CoV-2 detection. However, some weakness including high false negative rate (Kucirka et al., 2020) gradually appeared. Under such circumstances, comparison between the antibody test and RNA test were performed by researchers. For example, Pan Y et al. found promising detection capability of the immunochromatographic strip assay in RNA negative clinically diagnosed cases (Pan et al., 2020). Based on RNA negative cases and multiple tests data, we were also able to compare our ALLtest with the RNA test. By clinically confirmed cases from HBCDC, we showed a significant (p = 0.0018) higher detection rate from the ALLtest than the RNA test. A more comprehensive story was told by our time-series data. Depending on multiple testing data from two centers, we were able to compare ALLtest with RNA results along the infection process. Data from two centers gave overall consistent results. Along the infection process, the RNA test was the one with high positive rate within 5 days, but for long term infection we can no longer rely on RNA test (the highest positive rate was only around 75%). After 5 days of infection, the positive rate of antibody results quickly exceeded the RNA test and reached nearly 100% 10 days from onset. And after 10 days, there was still a stable higher positive rate of the ALLtest than the RNA test. As a long-last antibody, IgG showed a high and stable positive rate all along, even after 30 days of infection. IgM showed fluctuating positive rate of the infection and possessed a higher positive rate than the RNA test in the middle infection process stage (10-20 days). (**Figure 4**) Although data of SHAPHC and CQGWZX mostly agreed, there were differences in details which may be caused by patient source. As an international transportation hub, 29 out of 30 patients from SHAPHC were cases imported from overseas (Russia, USA, etc.), while all 40 patients from CQGWZX were indigenous. Conflicts between two centers mainly came from the data after 30 days of infection. CQGWZX showed evidence of a higher positive rate of IgG than RNA, but SHAPHC showed the same positive rate. After checking our data, we found CQGWZX possessed 10 samples which were much larger than the 4 samples in SHAPHC after 30 days of infection and thus, should be more reliable. The “75%” positive rate of IgG and RNA in SHAPHC was due to only one negative sample from an atypical patient, aged 76, with relatively mild symptoms. Therefore, based on HBCDC and CQGWZX data, we confirmed that the ALLtest was a good addition to the RNA test, especially for long-term infection. Despite the above strength, we still need to admit the limitation of our ALLtest. Unlike the RNA test which detected the current presence of virus, based on the complexity of immune mechanisms, it is hard to give exact results by antibody tests. Similar to other rapid antibody tests, the ALLtest cannot distinguish active infection from past infection which clearly requires caution in result interpretation.

Several studies have tried to find clinical factors correlated with antibody test results and several factors have been identified such as clinical severity (Zhao et al., 2020), and age (Irwin et al., 2021). With a large number of samples, even only based on a 0/1 result, we still could identify factors correlated with our ALLtest results and compare factors among centers. Probably due to a small number of confirmed COVID-19 patients, no correlated factor were identified for data from FAHZU. Disease course, the factor we care most about, was found to be correlated with IgM, IgG, and RNA results in HBCDC (**Table 4S**; **Figure 3**). IgM, IgG, and RNA were more likely to test positive during the late stage of disease onset. For the first time, we found that age correlated with both IgM and RNA results, however, in opposite ways as IgM positive patients tended to be older and RNA positive patients tended to be younger, (**Figures 3B, D**). In order to exclude the effect of disease course, we analyzed and confirmed no correlation between age and disease course (p = 0.12). A previous study using five rapid antibody tests on 102 clinically confirmed cases also found significantly lower IgM sensitivity in younger (under 40 years old) COVID-19 patients (Irwin et al., 2021). However, within all five tests, the only one with a non-significant result was the ALLtest. Here, using more than twice the number of cases (256), we found the IgM sensitivity of the ALLtest was also significantly higher in older patients (p = 0.00015). To explain the correlation between age and RNA test results, we searched for literature on age and viral load. However, contradictory results were found. Differences were observed but no significant viral-load difference among age groups was reported by several studies (Jacot et al., 2020; Kleiboeker et al., 2020; Owusu et al., 2020; Walsh et al., 2020). Partly agreeing with our result, Buchan B et al. (Buchan et al., 2020) found significantly lower CT values in the 80-89 age group. Inconsistent results were also reported by Euser S et al. (Euser et al., 2021) revealing that a young age group (<12 years) showed significantly lower viral loads. In our HBCDC cohort of 255 confirmed cases with age information, only 22 cases were under age 30 and only 2 cases were under age 20. Therefore, the above results may not be comparable with our data. For WHSYY data, we found sex (p = 0.017) and symptom (fever or not) (p = 0.012) were correlated with IgM results. Female COVID-19 patients and COVID-19 patients with fever symptom were more likely to be tested IgM positive. Accumulated evidence showed gender disparities in COVID-19 mortality in which males have a higher risk for worse outcomes including death (Jin et al., 2020). In line with such a fact, females develop higher immune responses towards both viral infection and vaccine (Ruggieri et al., 2016), which may due to the fact that estrogen and testosterone promotes and inhibits IgM, respectively (Kanda et al., 1996; Kanda and Tamaki, 1999). As a type of antibody being quickly expressed after infection, IgM plays a critical role in antiviral response. Although only by rapid antibody test, our results agreed with above findings. As for the symptoms, we tried to analyze between severity and antibody result but no significant correlation was found, which may be due to artificial classification. Therefore, we further divided confirmed cases into two groups based on an objective criterion (fever *vs*. non-fever). This time, significant correlation was found (patients with fever tended to be tested IgM positive) which agreed with previous results (positive correlation between severity and Ab titer) (Zhao et al., 2020).

## Conclusion

In summary, we deeply studied antibody results of the ALLtest from various aspects. We confirmed high reliability of our ALLtest and its important role in COVID-19 diagnosis. With a large number of samples, we further identified several correlating factors and those factors will require special attention during future clinical application.

## Data Availability Statement

The raw data supporting the conclusions of this article will be made available by the authors, without undue reservation.

## Ethics Statement

The studies involving human participants were reviewed and approved by Ethical Committee of the First Affiliated Hospital - Zhejiang University School of Medicine, Hubei Provincial Center for Disease Control and Prevention, The Third Hospital of Wuhan, Shanghai Public Health Clinical Center and Chongqing Public Health Medical Center.

## Author Contributions

YP analyzed the data, wrote the initial draft of the manuscript, and edited the manuscript. YHWeng and YHWu collected the data and performed the experiments. FG, XZ, and XX were responsible for the medical ethics and clinical trials. HL assisted in the completion of medical ethics. QK designed the study, reviewed the paper and administrated the project. All authors contributed to the article and approved the submitted version.

## Funding

This research was supported by National Natural Science Foundation of China (31501050), Zhejiang Provincial Program for the Cultivation of High-level Innovative Health Talents (WJW2021002), the Key R&D projects of Zhejiang province (2019C03057) and the Emergency Scientific Research Project on COVID-19 by the Collaborative Innovation Center of Yangtze River Delta Region Green Pharmaceuticals and the National Engineering Research Center for Process Development of Active Pharmaceutical Ingredients.

## Conflict of Interest

Author FG, XZ, and XX are employed by Hangzhou AllTest Biotech Co., Ltd.

The remaining authors declare that the research was conducted in the absence of any commercial or financial relationships that could be construed as a potential conflict of interest.

## Publisher’s Note

All claims expressed in this article are solely those of the authors and do not necessarily represent those of their affiliated organizations, or those of the publisher, the editors and the reviewers. Any product that may be evaluated in this article, or claim that may be made by its manufacturer, is not guaranteed or endorsed by the publisher.

## Abbreviation

FAHZU, The First Affiliated Hospital, Zhejiang University School of Medicine; HBCDC, Hubei Provincial Center for Disease Control and Prevention; WHSYY, The Third Hospital of Wuhan; SHAPHC, Shanghai Public Health Clinical Center; CQGWZX, Chongqing Public Health Medical Center.
