# Supplementary material for: Antibody Response to SARS-CoV-2 in the First Batch of COVID-19 Patients in China by a Self-Developed Rapid IgM-IgG Test
Source: Front Cell Infect Microbiol. 2022 May 27;12:915751. doi: 10.3389/fcimb.2022.915751 (PMC9204641; doi:10.3389/fcimb.2022.915751)
Supplement: Supplementary file 1 [file Table_1.docx]

Supplementary Material

Table 1S Sensitivity and specificity of ALLtest in preliminary test (FAHZU)

|  | Confirmed | Excluded |
| --- | --- | --- |
| **Sample size** | 22 | 10 |
| **IgM positive** | 17 | 0 |
| **IgG positive** | 22 | 1 |
| **IgM/IgG positive** | 22 | 1 |
| **Sensitivity** | 100% |  |
| **Specificity** |  | 90% |

Table 2S Sensitivity of ALLtest and qRT-PCR based on disease course in official test

|  | **FAHZU** | | | | | **HBCDC** | | | | | **WHSYY** | | | | |
| --- | --- | --- | --- | --- | --- | --- | --- | --- | --- | --- | --- | --- | --- | --- | --- |
| **Disease course (days)** | n | Ab |  | RNA |  | n | Ab |  | RNA |  | n | Ab |  | RNA |  |
|  |  | n(+) | sensitivity | n(+) | sensitivity |  | n(+) | sensitivity | n(+) | sensitivity |  | n(+) | sensitivity | n(+) | sensitivity |
| **1-7** | 0 | 0 | - | 0 | - | 90 | 81 | 90% | 67 | 74.44% | 18 | 17 | 94.44% | 18 | 100% |
| **8-14** | 0 | 0 | - | 0 | - | 100 | 94 | 94% | 80 | 80% | 9 | 9 | 100% | 8 | 88.89% |
| **15-21** | 1 | 1 | 100% | 1 | 100% | 23 | 23 | 100% | 23 | 100% | 29 | 27 | 93.10% | 29 | 100% |
| **22-28** | 0 | 0 | - | 0 | - | 16 | 16 | 100% | 16 | 100% | 31 | 31 | 100% | 31 | 100% |
| **>=28** | 63 | 61 | 96.83% | 63 | 100% | 26 | 22 | 84.62% | 26 | 100% | 117 | 115 | 98.29% | 117 | 100% |
| **In total** | 64 | 62 | 96.88% | 64 | 100% | 255 | 236 | 92.55% | 212 | 83.14% | 204 | 199 | 97.55% | 203 | 99.51% |

n(+): number of positive cases

Table 3S Results of ALLtest and qRT-PCR for all clinical confirmed cases in HBCDC

| IgM/IgG | qRT-PCR | | In total |
| --- | --- | --- | --- |
|  | + | - |  |
| + | 195 | 42 | 237 |
| - | 17 | 2 | 19 |
| In total | 212 | 44 | 256 |

Table 4S Factors correlation significance in FAHZU, HBCDC and WHSYY

| **P value** | **FAHZU** | | **HBCDC** | | | **WHSYY** | |
| --- | --- | --- | --- | --- | --- | --- | --- |
|  | IgM | IgG | IgM | IgG | RNA | IgM | IgG |
| Age | 0.66 | 0.97 | **0.00015** | 0.97 | **1.66e-06** | 0.32 | 0.22 |
| Sex | 1 | 0.56 | 0.70 | 0.80 | 0.80 | **0.017** | 0.74 |
| Disease course | 0.66 | 0.75 | **2.98e-06** | **0.034** | **3.09e-06** | 0.19 | 0.052 |
| Severity | 0.68 | 0.11 | - | - | - | 0.92 | 0.39 |
| Other disease | 0.36 | 0.22 | - | - | - | 1 | 1 |
| Symptom | 1 | 0.33 | - | - | - | **0.012** | 0.45 |
| Image | 0.57 | 1 | - | - | - | 0.87 | 0.18 |
| WBC | - | - | - | - | - | 0.85 | 0.74 |
| LYM | - | - | - | - | - | 0.75 | 0.74 |

Other disease: COVID-19 with only inflammation of the lungs and bronchial tubes is marked as N; COVID-19 with underlying diseases or complications that are unrelated to the respiratory system is marked as Y

Symptom: fever is marked as 1, others are marked as 0

Image: lung imaging features (normal is marked as 0; lung shadow is marked as 1; lung consolidation is marked as 2; other vague descriptions are not included)

WBC: Total number of white blood cells

LYM: Total number of lymphocytes

Table 5S Sex/Symptom vs. IgM in WHSYY

| IgM | Sex | | | Symptom | | |
| --- | --- | --- | --- | --- | --- | --- |
|  | male | female | Total | 1 | 0 | Total |
| + | 40 | 61 | 101 | 80 | 21 | 101 |
| - | 59 | 44 | 103 | 64 | 39 | 103 |
| Total | 99 | 105 | 204 | 144 | 60 | 204 |
